# Edward Gamalial Janeway

**Published:** 1911-03

**Authors:** Charles G. Stockton


**A Monthly Review of Medicine and Surgery**

*EDITOR*

WILLIAM WARREN POTTER, M. D.

All communications, whether of a literary or business nature, books for review and exchanges, should be addressed to the editor, 238 Delaware Avenue, Buffalo, N.Y.

Vol. Lxvi. MARCH, 1911. No. 8

### Edward Gamalial Janeway

On the evening of Friday, February 10, 1911, the wires throughout the land bore the announcement of the death, at Summit, N. J., of Dr. Edward Gamalial Janeway. There is scarcely an intelligent layman, no matter where residing, who failed to understand in this message that a renowned physician had passed away; but among this great number there was an unknown, yet relatively large proportion, who realised that they had suffered a personal loss. Probably no single American physician was so widely known and there was no other to whom so many have turned instinctively for judgment and decision, when such were needed for the welfare of a friend or the assistance of any individual whose existence was of public importance. The estimation in which Dr. Janeway was held is remarkable when it is recalled that his services were so largely confined to the routine duties of diagnostician, consultant and teacher. There was no extraneous element, no sensational note that brought him conspicuously before the world. His was a striking illustration of the fact that a man may acquire an enduring reputation when to ability there is joined sincerity.

He owed his power, in part, to that mysterious thing which we call personality, but in greater part to the fact that he was able to use inductive reasoning with a steadfast aim at high ideals in medicine. His experiences and achievements were those possible to one having, and availing himself of, the wide opportunities of a metropolitan physician. As to personality, his was not of the obtrusive kind. In fact, the simplicity and modesty of his demeanor were in strong contrast with his position and his accomplishments. A stranger seeing him in a general assembly noted the intelligence and earnestness of his face, but was struck by the air of detachment from the ordinary attractions of the occasion which his manner and conversation betrayed. He was a marked figure, even when unknown, but when his name was announced every one looked in his direction. Dr. Janeway's high place in the medical profession was not the result of his writings, although these were of a high order and worthy of the man, but his grasp upon the profession, as upon the public, was a more personal one and depended upon the fact that he had the gift of being right upon questions. He achieved a reputation as an anatomist and thereupon he acquired a still greater name as a pathologist. From these high planes and largely because of them he became an unsurpassed diagnostician and clinician. Many of his contributions were made to the Association of American Physicians, to which organization he was strongly attached, but all scientific bodies and philanthropic movements counted upon his loyal support.

It is impossible in the space permitted to write a critical review of his career, and beside it is not easy to review the life of Dr. Janeway. It might be summed up in the phraseology of pupil, teacher and practitioner, but under these headings there must be written countless subdivisions, and chief among these is this that he continually rendered service to all classes of humanity from the president of the nation to the humblest of its citizens.

CHARLES G. STOCKTON.

